# PRMT5 facilitates angiogenesis and EMT via HIF-1α/VEGFR/Akt signaling axis in lung cancer

**DOI:** 10.18632/aging.204826

**Published:** 2023-07-03

**Authors:** Yonghua Zheng, Huaxia Ji, Wulin Yi, Zhanjun Chen, Xiaobiao Hu, Jie Zhou, Yang Wang, Xiao Zheng

**Affiliations:** 1 Department of Pulmonary Medicine, Shanghai Jinshan Tinglin Hospital, Shanghai, P.R. China; 2 Dapartment of Pulmonary and Critical Care Medicine, Minhang Hospital, Fudan University, P.R. China; 3 Department of Emergency, The 8th People's Hospital of Shanghai, Shanghai, P.R. China

**Keywords:** PRMT5, HIF-1α, EMT, angiogenesis, lung cancer

## Abstract

Abnormal angiogenesis is a critical factor in tumor growth and metastasis, and protein arginine methyltransferase 5 (PRMT5), a prominent type II enzyme, is implicated in various human cancers. However, the precise role of PRMT5 in regulating angiogenesis to promote lung cancer cell metastasis and the underlying molecular mechanisms are not fully understood. Here, we show that PRMT5 is overexpressed in lung cancer cells and tissues, and its expression is triggered by hypoxia. Moreover, inhibiting or silencing PRMT5 disrupts the phosphorylation of the VEGFR/Akt/eNOS angiogenic signaling pathway, NOS activity, and NO production. Additionally, inhibiting PRMT5 activity reduces HIF-1α expression and stability, resulting in the down-regulation of the VEGF/VEGFR signaling pathway. Our findings indicate that PRMT5 promotes lung cancer epithelial-mesenchymal transition (EMT), which might be possibly through controlling the HIF-1α/VEGFR/Akt/eNOS signaling axis. Our study provides compelling evidence of the close association between PRMT5 and angiogenesis/EMT and highlights the potential of targeting PRMT5 activity as a promising therapeutic approach for treating lung cancer with abnormal angiogenesis.

## INTRODUCTION

Lung cancer is a prevalent and dangerous cancer that starts in the lungs and is one of the leading causes of cancer-related deaths worldwide, accounting for nearly one in five deaths caused by this cancer. Lung cancer can be divided into two main types: small-cell lung cancer (SCLC) and non-small cell lung cancer (NSCLC) [1–3]. NSCLC, which includes adenocarcinoma, squamous cell carcinoma, and large cell carcinoma, is the more common type of lung cancer, accounting for about 80-85% of all cases [4]. SCLC accounts for the remaining 15-20% of cases. Therefore, it is crucial to investigate the molecular mechanisms underlying lung cancer development to uncover potential therapeutic targets and improve lung cancer treatment in its early stages.

Angiogenesis is the process of forming new blood vessels from existing vessels. In normal physiology, angiogenesis is a natural and regulated process during growth, wound healing, and embryonic development [5, 6]. However, in human cancers, angiogenesis is often abnormal and uncontrolled. Cancer cells require a blood supply to grow and spread, and they stimulate the growth of new blood vessels to meet their metabolic needs [7]. This uncontrolled angiogenesis creates a network of blood vessels that nourish the cancer cells and supply oxygen and nutrients, enabling them to grow and spread to other parts of the human body. A growing body of evidence suggests that targeting angiogenesis is a promising strategy for cancer treatment [8]. Anti-angiogenic drugs work by inhibiting the growth of new blood vessels, thus limiting the blood supply to cancer cells [9]. In both types of lung cancer, angiogenesis plays a critical role in the development and progression. Thus, understanding the underlying mechanisms and regulating factors is crucial for developing effective cancer therapies.

The developing evidence suggests that methylation plays a crucial role in cancer development [10], and type II protein arginine methyltransferase 5 (PRMT5) can methylate histone or non-histone protein to govern RNA processing, chromatin stability, cell proliferation, survival, cell metabolism, and cancers, which is emerging as an attractive therapeutic target recently [11, 12]. PRMT5 has been shown to be overexpressed in several types of cancer and associated with a more aggressive phenotype and poorer prognosis. Recent studies have shown that PRMT5 is involved in developing and progressing various types of cancer, including breast, lung, prostate, colorectal, and gastric cancer, among others [12–17]. Moreover, studies have suggested that PRMT5 could be a potential target for developing anti-cancer therapies [18, 19], as inhibiting its activity has been shown to inhibit the growth of cancer cells and reduce tumor size. Nevertheless, it is still unclear whether PRMT5 regulates angiogenesis to promote lung cancer cell metastasis, and the potential molecular mechanism remains obscure.

Our study highlights the vital role of PRMT5 in the development and progression of lung cancer. By showing that PRMT5 was induced by hypoxia and participated in regulating angiogenesis, EMT, and metastasis, this study provided valuable insights into the molecular mechanisms underlying these processes in lung cancer.

## RESULTS

### PRMT5 is highly expressed in human lung cancer

**Figure 1:**
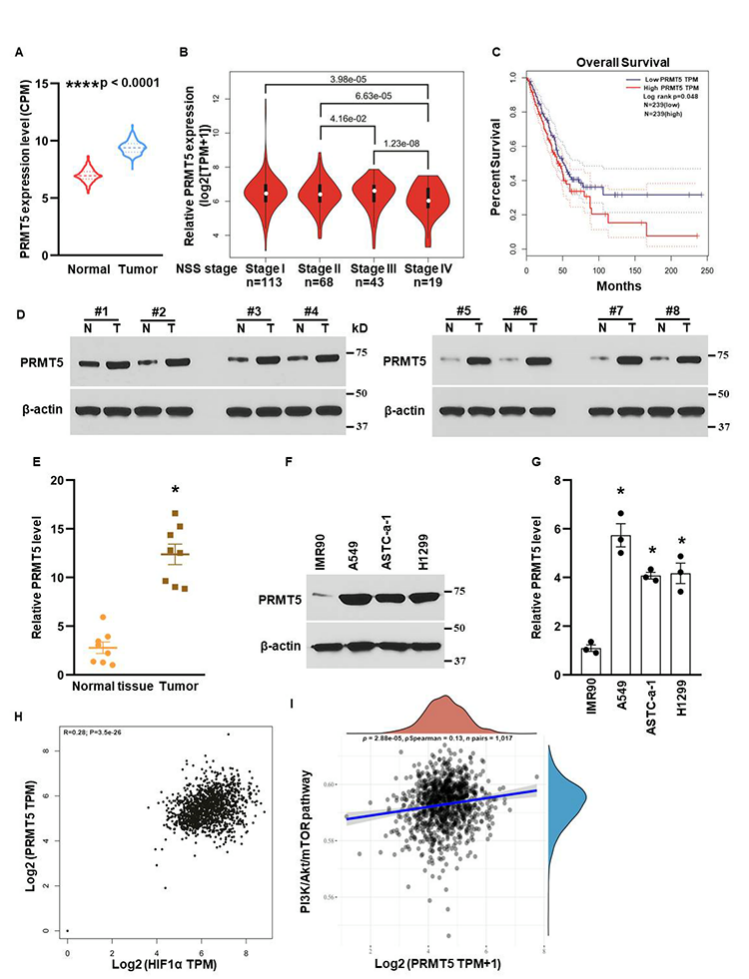
**Overexpression of PRMT5 results in human lung cancer progression.** (**A**) PRMT5 mRNA expression level was analyzed in adjacent normal tissues (n=486) and lung tumor tissues (n=338) using TCGA database. ****P <0.0001 vs. normal tissues with Mann-Whitney test. (**B**) PRMT5 was closely associated with stages in human lung cancer with analysis of the TCGA database. N=113 for stage I; n=68 for stage II; n=43 for stage III and n=19 for stage IV. P <0.001 vs. indicated stages. (**C**) PRMT5 expression level was negatively correlated with the patient’s overall survival with TCGA database analysis. n=239 for PRMT5-low group and PRMT5-high group. P values were determined by log-rank test and p=0.048. (**D**) PRMT5 protein expression levels were assessed by Western blotting in adjacent normal tissues (N) and lung tumor tissues (T). (**E**) PRMT5 protein expression levels were quantified in adjacent normal tissues and lung tumor tissues (n=8, each group). *P < 0.05 vs. normal tissue. (**F**) PRMT5 protein expression was detected by Western blotting in the indicated cell lines. (**G**) PRMT5 protein expression levels were quantified in the indicated cell lines. (n=3). *P < 0.05 vs. IMR90 cells. (**H**, **I**) The correlations between PRMT5 and HIF-1α or PI3K/Akt pathway were analyzed. The correlations between individual gene and pathway score were analyzed with Spearman method as well. The abscissa represents the distribution of the gene expression, and the ordinate represents the distribution of the pathway score. The density curve on the right represents the trend in distribution of pathway immune score; the upper density curve represents the trend in distribution of the gene expression. The value on the top represents the correlation p value, correlation coefficient and correlation calculation method.

**Figure 2:**
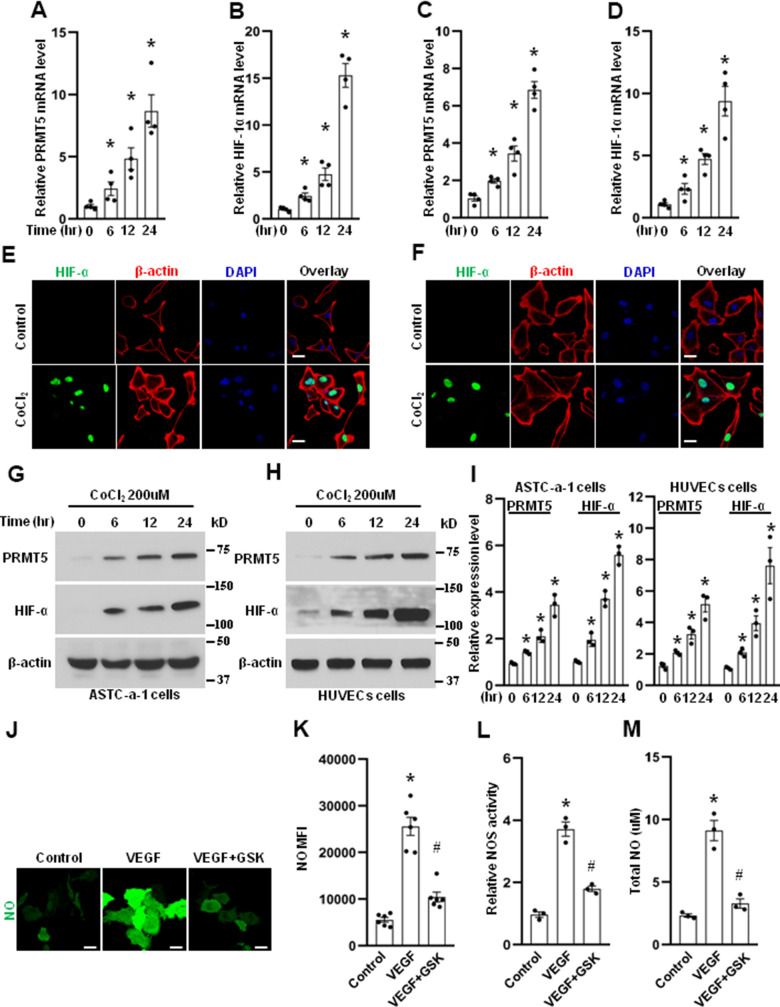
**PRMT5 is induced by hypoxia.** The HUVECs (**A**, **B**) and ASTC-a-1 cells (**C**, **D**) were treated with CoCl_2_ (200μM) for indicated time points, and the mRNA expression levels of PRMT5 and HIF-1α were measured by qRT-PCR. *P < 0.05 vs. 0-time point, n=4. The HUVECs (**E**) and ASTC-a-1 cells (**F**) were treated with or without CoCl_2_ (200μM) for 12h, and the HIF-1α expression induced by CoCl_2_ was detected by immunofluorescence staining. Representative pictures were shown. Green= HIF-1α; Red=β-actin; Blue=DAPI. Scale Bar=50μm. The ASTC-a-1 cells (**G**) and HUVECs (**H**) were treated with CoCl_2_ (200μM) for indicated time points, and the protein expression levels of PRMT5 and HIF-α were evaluated by Western blotting. (**I**) PRMT5 and HIF-α protein expression levels were quantified in ASTC-a-1 and HUVECs cells (n=3). *P < 0.05 vs. 0-time point. (**J**) The HUVECs were treated with VEGF (50ng/ml) in the presence or absence of GSK591, and the endothelial NO was monitored by the DAF-FM DA probe. Scale bar=50μm. (**K**) Quantitation of corresponding MFI values (n=6, each group). *P < 0.05 vs. control; ^#^P < 0.05 vs. VEGF. (**L**) NOS enzymatic activity in HUVECs upon treatment of VEGF with or without GSK591 as evaluated by the Griess method. *P < 0.05 vs. control; ^#^P < 0.05 vs. VEGF (50ng/ml) treatment. (n=3, each group). (**M**) Total NO was measured by ELISA kit using the Griess reaction in the supernatant of HUVECs upon treatment of VEGF (50ng/ml) with or without GSK591. *P < 0.05 vs. control; ^#^P < 0.05 vs. VEGF treatment. (n=3, each group).

To define the biological functions of PRMT5 in lung cancer, we first evaluated the relative expression levels of PRMT5 in lung tumor tissues and normal tissues. As seen in Figure 1A, PRMT5 mRNA expression was much higher in lung tumor tissues than in normal tissues, indicating that PRMT5 is up-regulated in lung cancer. Next, we assessed the association of PRMT5 expression with the lung tumor stages. As seen in Figure 2B, PRMT5 expression was much higher in stage II, stage III, and stage IV than in stage I, and the ectopic expression levels of PRMT5 were positively correlated with the stages. Furthermore, the patients with high expression levels of PRMT5 had a low survival rate compared with the patients with low expression levels of PRMT5 (Figure 1C). To validate the above findings, we collected the eight paired-lung tumor tissues and normal tissues from the patients with lung cancer and the PRMT5 protein expression were determined by Western blotting. As seen in Figure 1D and 1E, PRMT5 protein expression levels were significantly elevated (4.4 fold) in tumor samples compared with normal samples. Additionally, we detected the PRMT5 protein expression in lung cancer cell lines and normal human fetal lung fibroblast cells (IMR90). Consistent with the above lung cancer tissue results, PRMT5 was overexpressed in lung cancer cells than IMR90 cells (Figure 1F, 1G). Previous studies showed that HIF-1α and PI3K/Akt signaling pathway was closely related to angiogenesis, metastasis, and EMT [20, 21]. We next assessed the correlation between PRMT5 and HIF-1α or PI3K/Akt signaling pathway. As seen in Figure 1H, 1I, we found that PRMT5 positively correlated with HIF-1α and PI3K/Akt signaling pathway in lung cancer, suggesting a potential association between these genes and pathways. Taken together, our findings indicate that abnormal expression of PRMT5 is closely related to the lung tumor stages and survival rates in patients with lung cancer, and PRMT5 may regulate angiogenesis, metastasis, and EMT through HIF-1α and PI3K/Akt signaling pathway.

### PRMT5 is induced by hypoxia, and NO production is suppressed by PRMT5 inhibition

PRMT5 is considered as an oncogene and overexpressed in many human cancers, but the role of PRMT5 in angiogenesis is still unclear. To dissect the function of PRMT5 in angiogenesis during lung cancer development, human umbilical vein ECs (HUVECs) were used in our study because HUVECs are a widely used cell model for studying vasculature and angiogenesis, and HUVECs also express many essential signaling molecules and endothelial markers associated with angiogenesis [22, 23]. We first determined the mRNA expression level of PRMT5 in HUVECs induced by CoCl_2_ that mimics hypoxia in different *in vitro* studies. As seen in Figure 2A, PRMT5 mRNA expression was dramatically increased, induced by CoCl_2_ in a time-dependent manner. Moreover, the HIF-1α, a marker for angiogenesis under hypoxia, was also markedly increased (Figure 2B). Hypoxia is one of the hallmarks of human lung cancer [24–26]. We wonder if the expression of PRMT5 and HIF-1α was changed in lung cancer cells induced by CoCl_2_. To address this question, the ASTC-a-1 cells, a lung adenocarcinoma cell line, were used. As seen in Figure 2C, 2D, both PRMT5 and HIF-1α mRNA levels were significantly elevated in ASTC-a-1 cells in a time-dependent manner upon CoCl_2_ treatment. In addition, we found that HIF-1α was highly expressed in the nucleus in both HUVECs (Figure 2E) and ASTC-a-1 cells (Figure 2F) in response to CoCl_2_ treatment. Next, PRMT5 and HIF-1α protein expression was determined in HUVECs and ASTC-a-1 cells induced by CoCl_2_. As seen in Figure 2G–2I, we found that PRMT5 and HIF-1α protein expression was increased in both HUVECs and ASTC-a-1 cells in a time-dependent manner.

NO plays an essential role in angiogenesis [27]. Thus, to determine whether PRMT5 is involved in NO production, the HUVECs were treated with VEGF in the presence or absence of GSK591, a potent PRMT5 inhibitor, and the NO production was monitored by the DAF-FM DA probe in living cells. As seen in Figure 2J, 2K, we found that NO production was strongly induced by VEGF compared with the control group, whereas NO production was markedly diminished by PRMT5 inhibitor GSK591. To further confirm whether PRMT5 is involved in NO production, the eNOS activity and total NO production in the medium of HUVECs were measured. As seen in Figure 2L, 2M, we found that both eNOS activity and total NO production were strongly induced by VEGF compared with the control group, whereas both eNOS activity and total NO production were lessened by PRMT5 inhibitor GSK591. Collectively, these results suggest that PRMT5 is engaged in NO production and angiogenesis, which may lead to EMT and metastasis during lung cancer progression.

### Blocking PRMT5 impairs VEGFR2/Akt/eNOS signaling axis

**Figure 3:**
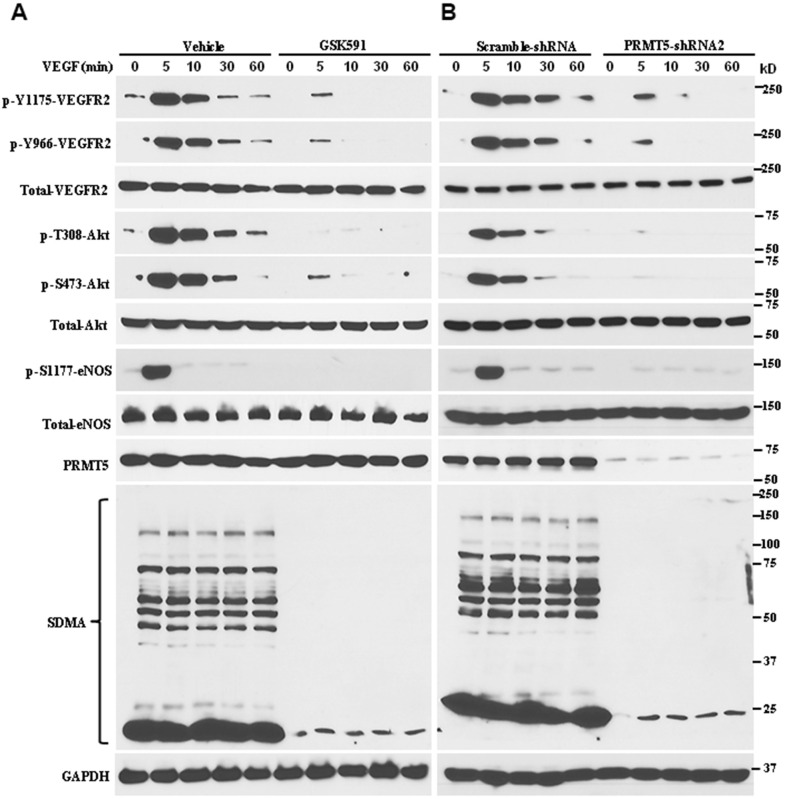
**Inhibiting PRMT5 impairs VEGFR2/Akt/eNOS signaling axis.** (**A**) The HUVECs were incubated with vehicle or GSK591 (10μM) for five days, and the cells were serum starved and treated with VEGF (50ng/mL) at different time points. The cells were harvested, and the indicated protein expression levels were assessed by Western blotting (n=3). GAPDH served as an internal control. (**B**) The HUVECs were infected with lentivirus containing scramble-shRNA or PRMT5-shRNA2, and the cells were serum starved. The cells were stimulated with VEGF (50 ng/mL), and the indicated protein expression levels were assessed by Western blotting (n=3). The PRMT5 activity was determined by the expression of SDMA.

VEGF/VEGFR2 signaling cascades, one of the most prominent angiogenetic factors, serve as an essential mediator to facilitate endothelial cell proliferation, survival, and migration, which promote abnormal angiogenesis. In order to dissect whether PRMT5 regulates angiogenesis and the possible molecular mechanism, we used the potent PRMT5 inhibitor to repress the PRMT5 enzyme activity. For this purpose, the HUVECs were treated with GSK591 for five days and then were stimulated with VEGF (50ng/ml) at indicated time points. As seen in Figure 3A, GSK591 treatment dramatically reduced the phosphorylation of VEGFR2 at both Tyr-1175 and Tyr966 induced by VEGF, and the symmetric dimethylation of arginine (SDMA) that was catalyzed by PRMT5 on a wide variety of targets was also reduced. In contrast, the total VEGFR2 was unchanged, indicating that blocking PRMT5 enzyme activity only affects the phospho-VEGFR2, but not the total protein. Furthermore, blocking PRMT5 also impaired the phosphorylation of Akt at both Thr-308 and Ser-473 and the phosphorylation of eNOS at Ser-1177 induced by VEGF, which were mainly engaged in endothelial cell duplication and angiogenesis. To further confirm whether PRMT5 regulates VEGFR2/Akt/eNOS signaling axis, we used lentivirus containing PRMT5-shRNA to knock down endogenous PRMT5, and the phosphorylation levels of VEGFR2, Akt, and eNOS were detected by Western blotting. As seen in Figure 3B, we found that down-regulation of PRMT5 attenuated the phosphorylation levels of VEGFR2, Akt, and eNOS induced by VEGF, similar to the GSK591 treatment. Altogether, these data indicate that PRMT5 indeed regulates angiogenesis via VEGFR2/Akt/eNOS signaling axis, which is related to PRMT5 enzyme activity.

### Inhibiting PRMT5 attenuates HIF-1α expression and stability induced by hypoxia

**Figure 4:**
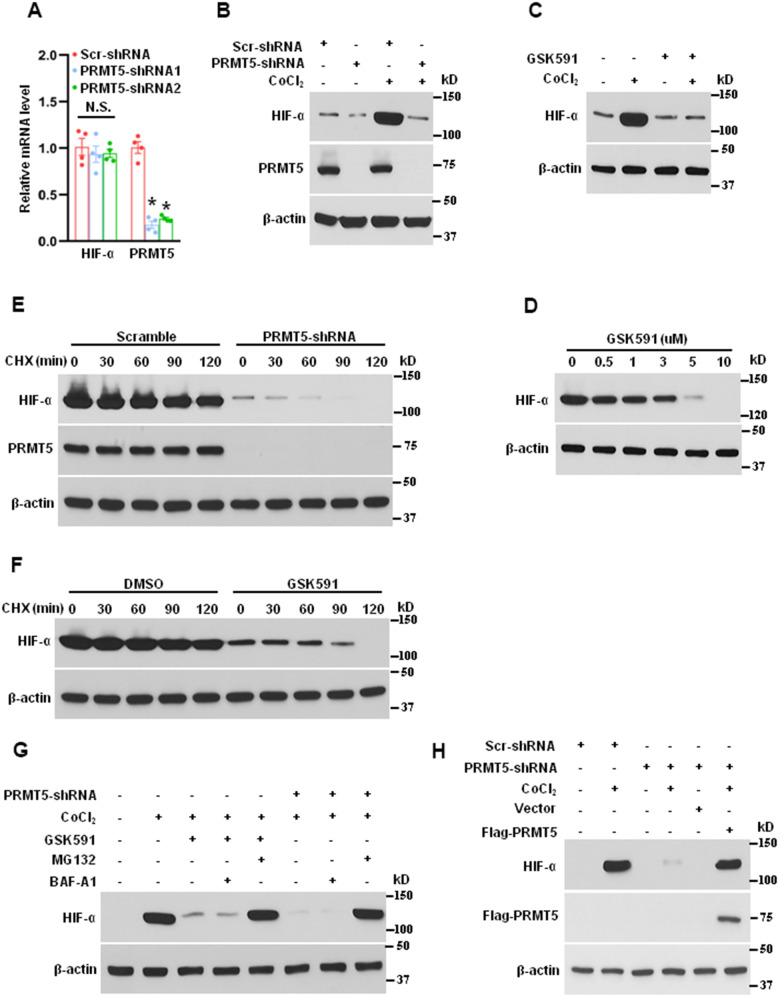
**Suppressing PRMT5 reduces the expression and stability of HIF-1α induced by hypoxia.** (**A**) The mRNA expression of HIF-1α and PRMT5 was measured by qRT-PCR in PRMT5 depletion cells (HUVECs, n=4). *P < 0.05 vs. Scr. N.S. means not significant. (**B**) The HUVECs were infected with lentivirus containing scramble-shRNA or PRMT5-shRNA2, followed by CoCl_2_ (200μM) treatment for 24h. The PRMT5 and HIF-α expression levels were determined by Western blotting (n=3). (**C**) The HUVECs were treated with vehicle or GSK591 (1μM) for five days and then were treated with CoCl_2_ (200μM) for 24h. The HIF-1α expression level was determined by Western blotting (n=3). (**D**) The effect of different doses of GSK591 on the HIF-1α expression level induced by CoCl_2_ as evaluated by Western blotting (n=3). (**E**) The HIF-1α stability was detected by Western blotting in PRMT5 depletion HUVECs upon cycloheximide (CHX, 20μg/mL) treatment at the indicated time points (n=3). (**F**) The HIF-1α stability was detected by Western blotting in GSK591-treated HUVECs upon cycloheximide treatment at the indicated time points (n=3). (**G**) The HUVECs were incubated with or without GSK591 (10μM) or infected with lentivirus containing PRMT5-shRNA2 and then pretreated with MG132 (10μM) and BAF-A1 (100nM) for 30 min before treating CoCl_2_. The HIF-1α expression levels were evaluated by Western blotting (n=3). (**H**) The HUVECs were infected with lentivirus containing scramble-shRNA or PRMT5-shRNA2 and then were transfected with vector or Flag-PRMT5 before treating CoCl_2_. The HIF-1α expression levels were evaluated by Western blotting (n=3).

HIF-1α is a heterodimeric transcription factor that plays a critical role in angiogenesis in response to ischemia or hypoxia [28]. To explore the potential function of HIF-1α and PRMT5 in lung cancer, we first evaluated the mRNA expression of HIF-1α in PRMT5 depletion cells. As seen in Figure 4A, down-regulation of PRMT5 did not affect the mRNA expression of HIF-1α, suggesting that a posttranslational mechanism may be involved in regulating HIF-1α protein levels by PRMT5. We next investigated whether down-regulation or inhibition of PRMT5 affects the protein expression of HIF-1α upon hypoxia stimulation. As seen in Figure 4B, 4C, both down-regulation and inhibition of PRMT5 strongly reduced the HIF-1α expression induced by CoCl_2_. Moreover, the reduction of HIF-1α expression was negatively associated with the concentration of GSK591 (Figure 4D). These findings imply that PRMT5 may control HIF-1α expression via a posttranslational mechanism. To further explore how PRMT5 regulates HIF-1α expression and the related molecular mechanism, we detected the HIF-1α expression stability in PRMT5 depletion HUVECs or treatment of GSK591 in the presence of cycloheximide (CHX). As seen in Figure 4E, 4F, down-regulation or inhibition of PRMT5 markedly decreased the stability of HIF-1α protein in response to CoCl_2_ and promoted its degradation. In order to understand which pathway mediated the degradation of HIF-1α, we used the bafilomycin A1 (BAF-A1), the lysosome inhibitor, and MG132, the proteasome inhibitor, to evaluate the HIF-1α degradation upon GSK591 treatment or in PRMT5 depletion HUVECs. As seen in Figure 4G, we found that HIF-1α degradation was entirely blocked by MG132 but not BAF-A1, indicating that PRMT5 regulated HIF-1α stability via the proteasome pathway, but not lysosome pathway. Finally, we wonder whether the reintroduction of PRMT5 could rescue the HIF-1α degradation in PRMT5 depletion HUVECs. To this end, we reintroduced WT-PRMT5 into the PRMT5 depletion HUVECs, and the cells were treated with CoCl_2._ As seen in Figure 4H, the reintroduction of WT-PRMT5 rescued the HIF-1α expression in PRMT5 depletion HUVECs compared with vector control induced by CoCl_2_. Collectively, our findings uncover a new mechanism by which PRMT5 promotes angiogenesis by regulating HIF-1α stability.

### Inhibiting PRMT5 attenuates EMT and metastasis in lung cancer

**Figure 5:**
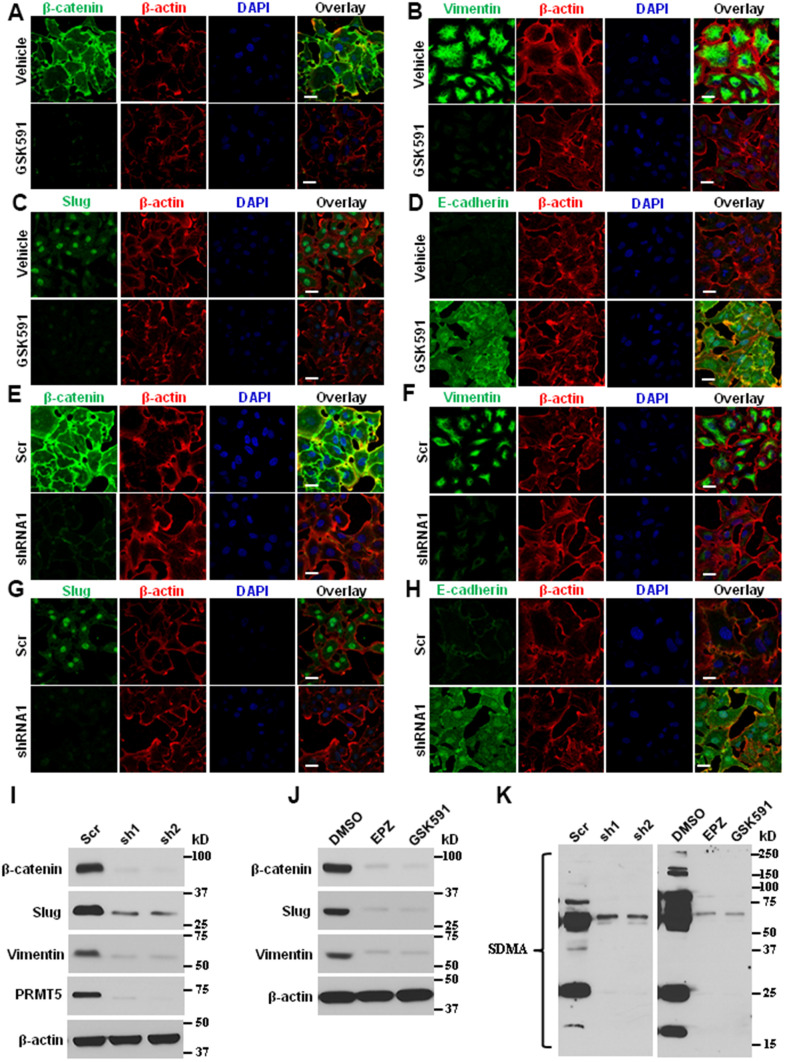
**Blocking PRMT5 attenuates lung cancer invasion and metastasis.** (**A**–**D**) The ASTC-a-1 cells were treated with GSK591 or Vehicle, and the EMT markers (β-catenin, Vimentin, Slug, and E-cadherin) were determined by immunofluorescence. Representative pictures were shown (n=3, each group). Scale Bar=50μm. (**E**–**H**) The ASTC-a-1 cells were infected with lentivirus containing scramble-shRNA or PRMT5-shRNA1, and the EMT markers (β-catenin, Vimentin, Slug, and E-cadherin) were determined by immunofluorescence. Representative pictures were shown (n=3, each group). Scale Bar=50μm. (**I**) The ASTC-a-1 cells were infected with lentivirus containing scramble-shRNA, PRMT5-shRNA1, or PRMT5-shRNA2. The expression levels of EMT markers (β-catenin, Vimentin, and Slug) and PRMT5 knockdown efficiency were detected by Western blotting (n=3). (**J**) The ASTC-a-1 cells were treated with GSK591, and the expression levels of EMT markers (β-catenin, Vimentin, and Slug) were detected by Western blotting (n=3). (**K**) The ASTC-a-1 cells were treated with GSK591 or Vehicle or infected with lentivirus containing scramble-shRNA, PRMT5-shRNA1, or PRMT5-shRNA2. The PRMT5 enzyme activity was detected by Western blotting (n=3) using the SDMA antibody.

Angiogenesis is a critical component of metastatic signaling and EMT by recruiting new blood vessels, which provides the principal route to facilitate the tumor cells to secede from the primary tumor site and enter circulation [29]. To probe the molecular basis of EMT and metastasis in lung cancer cells mediated by PRMT5, the ASTC-a-1 cells were treated with PRMT5 inhibitor GSK591 or infected with lentivirus expressing PRMT5-shRNA, and the EMT markers were monitored by immunofluorescence. As seen in Figure 5A–5D, inhibition of PRMT5 significantly repressed the expression of β-catenin, Vimentin, and Slug, whereas the expression of E-cadherin was increased upon GSK591 treatment. Similar results were obtained in PRMT5 depletion cells (Figure 5E–5H). To confirm the above findings, these markers were also detected by Western blotting. As seen in Figure 5I and 5J, the expression levels of β-catenin, Vimentin, and Slug were diminished in response to PRMT5-specific inhibitors GSK591 and EPZ or down-regulation of PRMT5. The PRMT5 enzyme activity was also reduced, indicated by the expression of SDMA (Figure 5K). Altogether, our findings suggest that PRMT5 regulates lung cancer cell EMT and metastasis, at least in part, via promoting angiogenesis by controlling HIF-1α/VEGFR/Akt/eNOS signaling pathway.

## DISCUSSION

**Figure 6:**
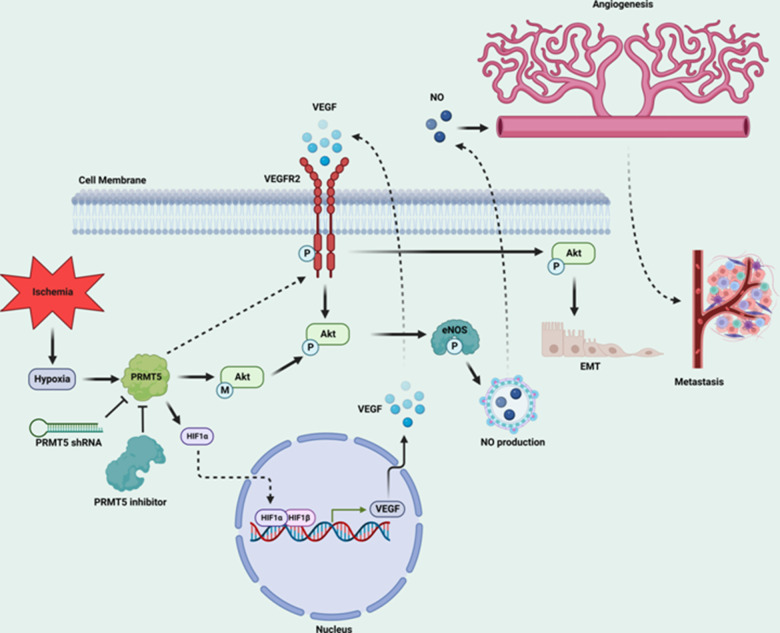
Graphic summary of the mechanism by which PRMT5 regulates HIF-1α expression and VEGFR2/Akt/eNOS to promote angiogenesis and metastasis in human lung cancer.

PRMT5, an oncoprotein, has been implicated in various stages of human cancer progression and development by altering signaling pathways [19, 30]. The exact role of PRMT5 in controlling cancer cell metastasis, particularly in human lung cancer, remains unclear. Our study showed that PRMT5 was highly expressed in human lung cancer tissues and correlated with the progression of lung tumors and patient survival rates (Figure 1). These results suggest that PRMT5 plays a key role in lung cancer progression. Additionally, our results showed that hypoxia-induced PRMT5 expression and inhibiting or silencing PRMT5 impeded the phosphorylation of VEGFR/Akt/eNOS angiogenic signaling axis and decreased eNOS activity and NO production (Figures 2, 3). PRMT5 inhibition or down-regulation also decreased HIF-1α expression and stability (Figure 4), resulting in inhibition of the VEGF/VEGFR signaling axis. Additionally, inhibiting or silencing PRMT5 also suppressed EMT markers in lung cancer cells (Figure 5). These findings suggest that PRMT5 promotes EMT and metastasis, at least partly, by governing HIF-1α/VEGFR/Akt/eNOS signaling and promoting angiogenesis (Figure 6).

PRMTs, a family of enzymes that catalyze the methylation of histone and non-histone proteins, have been recognized for their crucial role in regulating various cellular processes, including chromatin regulation, signal transduction, RNA processing, and gene expression [9, 26]. PRMT5, a major type II methyltransferase, is widely expressed in human cancers and has a crucial role in cancer development [16, 31]. However, the function of PRMT5 in angiogenesis and lung cancer EMT is still largely unknown. The evolving evidence suggests that the role of PRMT5 in cancer development has recently gained significant attention. An increase in PRMT5 expression has been found to be closely linked to the occurrence and progression of various types of tumors. It has been reported that PRMT5 overexpression promoted tumor growth, while depletion of PRMT5 inhibited tumor growth [12]. The inhibition of PRMT5 can increase the number of infiltrating immune cells and improve antitumor immunity by reducing the expression of NLRC5 and methylating IFI116/IFI204 [32]. High levels of PRMT5 expression in lung cancer have been associated with poor prognosis, and inhibition of PRMT5 can affect the growth cycle of lung cancer cells by inhibiting the phosphorylation of AKT1 [12, 15]. PRMT5 has also been shown to promote metastasis of lung cancer cells by activating the AKT1 and ERK signaling pathways [33, 34]. Our study has revealed that PRMT5 was not only highly expressed in lung tumors but also closely linked with poor prognosis and survival rate. Despite the increasing recognition of PRMT5’s significance in tumors, its role in angiogenesis and EMT is entirely unknown.

Hypoxia is a common phenomenon in solid tumors, including lung cancer, where there is a lack of oxygen supply to the tumor cells [35, 36]**.** This lack of oxygen leads to metabolic changes in the tumor cells and affects various cellular processes, such as angiogenesis, cell migration, and invasion [37, 38]. These changes contribute to the aggressive behavior of the tumor and the development of resistance to cancer treatments. Hypoxia has also been shown to play a crucial role in regulating genes involved in tumor progression, such as VEGF, HIF-1α, and c-Myc, leading to the promotion of angiogenesis, cell growth, and invasion [38–41]. In addition, hypoxia can promote the development of cancer stem cells, which are cells within a tumor that have the ability to give rise to all the different cell types found in the tumor and are often resistant to cancer treatments [41]. The management of hypoxia in lung cancer is a major challenge in treating the disease. Moreover, the HIF-1α/VEGFR/Akt/eNOS signaling axis is upregulated under hypoxic conditions in tumors and contributes to cancer development and progression by promoting angiogenesis and EMT of tumor cells [20, 42]. HIF-1α activates VEGF to stimulate endothelial cell proliferation, migration, and survival, while activation of the Akt pathway downstream of VEGFR promotes EMT. HIF-1α also activates eNOS expression, leading to the production of NO, which promotes angiogenesis [43]. Additionally, NO, produced by endothelial nitric oxide synthase (eNOS), can be activated by various stimuli, including HIF-1α, and also promotes tumor angiogenesis by inducing endothelial cell growth, migration, and tube formation [44, 45]. Therefore, up-regulation of the HIF-1α/VEGFR/Akt/eNOS signaling axis, which activates eNOS expression, can increase NO production and promotion of tumor angiogenesis. Targeting this signaling axis may be a potential therapeutic strategy for cancer treatment. Our findings revealed that PRMT5 was induced by hypoxia, whereas inhibiting or silencing PRMT5 impaired the phosphorylation of the VEGFR/Akt/eNOS angiogenic signaling axis and decreased eNOS activity and NO production. Also, suppressing PRMT5 attenuated HIF-1α expression and stability induced by hypoxia, which led to down-regulation of the phosphorylation of the VEGF/VEGFR2 signaling axis. These results indicate that PRMT5 is a central upstream mediator for angiogenesis under hypoxia and imply that PRMT5 is a therapeutic candidate for treating lung cancer with abnormal angiogenesis. The current treatments aim to improve oxygen delivery to the tumor, such as oxygen inhalation therapy or angiogenic agents to enhance blood flow to the tumor. Others aim to target the hypoxia-related pathways and genes, such as using HIF-1α inhibitors. However, further research is needed to fully understand the role of PRMT5 in lung cancer under hypoxia and to develop effective treatments under this condition.

PRMT5 has been implicated in regulating EMT, a biological process through which epithelial cells lose their cell-cell adhesion and acquire mesenchymal cell properties, resulting in increased migration, invasion, and stemness [18, 19]. During EMT, epithelial cells undergo changes in gene expression, cell-cell adhesion, and cytoskeleton organization, which promote the loss of cell polarity, increased cell motility and invasiveness, and a more mesenchymal-like phenotype. Studies have shown that PRMT5 regulates EMT by modifying various target proteins, including transcription factors and cell adhesion molecules [15]. For example, PRMT5 has been shown to regulate Snail expression and activity, a transcription factor that is a key regulator of EMT [12]. PRMT5 has also been shown to directly methylate Akt1 arginine 15, a master regulator for cancer metastasis, leading to cancer cell invasion and migration [12]. Our findings present solid proof of a strong association between PRMT5 and angiogenesis/EMT and imply that inhibiting PRMT5 activity could be a viable therapeutic strategy for combating lung cancer that exhibits abnormal angiogenesis. Further studies are needed to fully understand the mechanism by which PRMT5 regulates angiogenesis and EMT and its potential as a therapeutic target in lung cancer treatment.

## MATERIALS AND METHODS

### Clinical materials collection

The matched adjacent normal tissues and related lung tumor tissues were collected between January 2020 and January 2022 from patients who were accepted with surgical treatment at Minhang Hospital of Fudan University. A total of eight matched tissue samples were collected and were immediately flash-frozen in liquid nitrogen. Then, those samples were used for further experiments and assays.

### Bioinformatics analysis

Bioinformatics analysis was performed with public datasets in TCGA and described previously [46]. We acquired TCGA datasets from Xena Browser. Gene expression dataset across normal and cancer tissue was extracted and analyzed. RNA expression values were transformed using the ‘voom’ method limma package, version 3.30.13. All multiple comparisons for TCGA analysis were corrected using Benjamini-Hochberg unless otherwise stated. For a direct comparison of the expression of PRMT5 between normal tissue and cancer tissues, an unpaired t-test or Mann-Whitney test was used, and p-values were calculated. TMM normalized counts were Log2 + 1 transformed and were used to classify patients into high and low expression groups for each gene of interest using the surv_cutpoint function in survminer R; then, four stages were generated from these data. The prognosis of each group was examined using Kaplan-Meier survival estimators with the survminer R package, version 0.4.1, with survival outcomes compared by log-rank tests. Moreover, RNA-sequencing expression (level 3) profiles and corresponding clinical information for PRMT5, HIF-1α and PI3K/Akt were downloaded from the TCGA dataset (https://www.cancer.gov/ccg/research/genome-sequencing/tcga). R software GSVA package was used to analyze, choosing parameter as method=‘ssgsea’. The correlation between genes and pathway scores was analyzed by Spearman correlation. All the analysis methods and R packages were implemented by R version 4.0.3. p-value <0.05 was considered statistically significant.

### Cell culture and inhibitors

The ASTC-a-1 cells were obtained from the Department of Medicine, Jinan University (Guangzhou, China), and the cells were cultured in Dulbecco’s Modified Eagle’s Medium (1:1) (DMEM, Gibco, Thermo Fisher Scientific) supplemented with 10% (v/v) fetal bovine serum (FBS, Sigma cat# F2442), 50 units/ml penicillin, and 50 mg/ml streptomycin. The Human umbilical vein endothelial cells (HUVECs) were purchased from Thermo Fisher Scientific (cat# C0035C), and the cells were cultured in Endothelial Cell Growth Medium (EGM-2 BulletKit, Lonza, cat# CC-3162) supplemented with 10% FBS, L-glutamine, and 1% antibiotic solution. All other cell lines were purchased from The Cell Bank of Type Culture Collection of Chinese Academy of Sciences (CAS) in Shanghai and were cultured in Dulbecco’s Modified Eagle’s Medium (DMEM, Gibco, Thermo Fisher Scientific) supplemented with 10% fetal bovine serum. The mycoplasma was measured by PlasmoTest™-Mycoplasma Detection Kit (InvivoGen, China) in ASTC-a-1cells and HUVECs before the experiments were performed. All the cells were maintained at 37° C with 5% CO_2_ in an incubator. The ASTC-a-1 cells and HUVECs were treated with 200μM Cobalt Chloride (CoCl_2_) for indicated time points to generate the hypoxic cell model. PRMT5 specific inhibitor EPZ015666 (GSK3235025, cat# HY-12727) and MG-132 (HY-13259) was purchased from MedChemExpress (MCE). The cycloheximide (cat# C4859) and GSK591 were purchased from Sigma (cat# SML-1751). The lysosome inhibitor bafilomycin A1 (BAF-A1, cat# 1334) was purchased from TOCRIS.

### Construction of plasmids

The Scramble-shRNA and PRMT5-shRNAs were described previously [30]. Briefly, to knock down endogenous PRMT5, the Scramble-shRNA and PRMT5-shRNAs were cloned into a lentiviral vector. The human PRMT5 targeting sequences were: shRNA1, 5’-GGATAAAGCTGTATGCTGT-3’; shRNA2: 5’-GCCATCTATAAATGTCTGCTA-3’. To generate the lentivirus containing Scramble-shRNA or PRMT5-shRNAs, the helper plasmids MD2G and PAX2 were used (Addgene). The human PRMT5 cDNA was subcloned into the Flag vector and verified by sequencing.

### Generation of PRMT5 stable knockdown cell line

The lentiviral constructs containing scramble-shRNA or PRMT5-shRNAs were co-transfected along with the helper plasmids MD2G and PAX2 into 293T cells using Lipofectamine™ 3000 (cat# L3000015, Invitrogen) transfection reagent according to the manufacturer’s protocol. After 24 h post-transfection, the culture media were replaced with fresh media, and then the culture media were harvested after 48 h post-transfection. The media were filtered, and the viral titer was pre-determined. Subsequently, ASTC-a-1 cells and HUVECs were infected with the lentivirus containing scramble-shRNA or PRMT5-shRNAs with an equal amount of virus particles. After 24 h, post-infection, the cells were selected with puromycin (Sigma, cat# p9620) for 48 h.

### Gene expression analysis

Gene expression was performed by quantitative real-time PCR (qRT-PCR). The total RNA was extracted from ASTC-a-1 cells and HUVECs upon treatments using TRIzol reagent (Invitrogen, cat# 15596-018) according to the manufacturer’s protocol. After extraction of the total RNA, the concentration of the RNA was measured by NanoDrop 2000 spectrophotometers, and the equal amount of RNA (1ug) was applied to carry out the reverse transcription by C1000 Touch PCR Thermal Cycler (Bio-Red). Finally, the qRT-PCR was performed to detect the gene expression with SYBR green fluorescent Dye (Bio-Rad, cat# 1725272) by an ABI7500 PCR machine (Applied Biosystems™). The following primers were used in this study: human PRMT5 forward: 5’-CCTGTGGAGGTGAACACAGT-3’ and revise: 5’-AGAGGATGGGAAACCATGAG-3’; human HIF-1α, forward: 5′-CACCTCTTTTGGCAAGCATCCTG-3′ and revise: 5′-TATGAGCCAGAAGAACTTTTAGGC-3′; human GAPDH, forward: 5′-TGTGGGCATCAATGGATTTGG-3′ and revise: 5′-ACACCATGTATTCCGGGTCAAT-3. GAPGH served as an internal control. The relative mRNA expression level was calculated by the method of ΔΔ-Ct.

### Western blot analysis

Western blot analysis was performed as described previously [47]. Briefly, the total proteins were extracted from tissues and cells with the lysis buffer (20 mmol/L Tris, PH 7.4, two mmol/L EDTA, two mmol/L EGTA, one mmol/L sodium orthovanadate, 1% Triton X-100, 150 mmol/L NaCl, 50 mmol/L sodium fluoride, 0.1% SDS, and 100 mmol/L phenylmethylsulfonyl fluoride) and then were centrifuged for 10 min at 4° C with the top speed of the centrifuge. Next, the Bradford method determined the protein concentration before separating the proteins in sodium dodecyl sulfate/polyacrylamide gel electrophoresis (SDS/PAGE). The proteins were transferred to the PVDF membranes (cat#1620177; Bio-Rad), and the membranes were washed three times with TBST and blocked using 5% non-fat milk for one h at room temperature. Next, the membranes were incubated with the indicated antibodies for overnight at 4° C: PRMT5 (Santa Cruz Biotechnology, cat# sc-376937), HIF-1α (Cell Signaling Technology, cat# 36169), Phospho-Tyr1175-VEGF Receptor 2 (Cell Signaling Technology, cat# 3770), phospho-Tyr996-VEGF Receptor 2 (Cell Signaling Technology, cat# 2474), total VEGFR 2 (Cell Signaling Technology, cat# 9698), phospho-Thr308-Akt (Cell Signaling Technology, cat# 4056), phospho-Ser473-Akt (cat# 4060; Cell Signaling Technology), total Akt (Cell Signaling Technology, cat# 4691), phospho-Ser1177-eNOS (Cell Signaling Technology, cat# 9570), total eNOS (Cell Signaling Technology, cat# 32027), Symmetric Di-Methyl Arginine Motif [sdme-RG] MultiMab™ (cat# 13222; Cell Signaling Technology), β-catenin (Cell Signaling Technology, cat# 8814), vimentin (Cell Signaling Technology, cat# 5741), Slug (Cell Signaling Technology, cat# 9585), Flag (Sigma, cat# F7425), β-actin (Santa Cruz Biotechnology, cat# sc-47778), and GAPDH (Cell Signaling Technology, cat# 5174). Then, the membranes were incubated with goat anti-rabbit conjugated to HRP secondary antibody (Santa Cruz Biotechnology, cat# sc-2004) or goat anti-mouse conjugated to HRP secondary antibody (Santa Cruz Biotechnology, cat# sc-2005) at room temperature for 2h. Finally, the proteins were detected by SuperSignal West Pico Chemiluminescent Substrate Western blotting reagents (Thermo Fisher Scientific, cat# 34580).

### Immunofluorescence

ASTC-a-1 cells were seeded into 6-well plates for 24h before treatments. The cells were fixed for 20 min at room temperature, and then permeabilization was applied with ice-cold methanol at -20° C for 10 minutes. The cells were washed with PBS twice after permeabilization and incubated in blocking buffer (5% normal goat serum) for 60 min at room temperature, followed by incubation with the primary antibodies: HIF-1α (Cell Signaling Technology, cat# 36169), β-catenin (Cell Signaling Technology, cat# 8814), vimentin (Cell Signaling Technology, cat# 5741), Slug (Cell Signaling Technology, cat# 9585), E-Cadherin (Cell Signaling Technology, cat# 3195) and β-actin (Santa Cruz Biotechnology, cat# sc-47778) (diluted 1:50 in blocking buffer) at 4° C overnight. After incubation, the cells were washed with PBS three times, followed by incubation with Alexa Fluor 594-conjugated goat anti-mouse secondary antibody (Thermo Fisher, cat# A-11005) and Alexa Fluor 488-conjugated goat anti-rabbit secondary antibody (Thermo Fisher, cat# A-11034). Finally, the nuclei were labeled with DAPI (Sigma, cat# D9542) before observation. The images were analyzed with a confocal microscopy system (LSM700, Zeiss).

### Nitric oxide synthase (NOS) activity measurement

To measure the NOS activity, HUVECs were treated with VEGF, GSK591, or vehicle, and the NOS activity was detected with the NOS activity assay kit (Abcam, ab211083) according to the manufacturer’s protocol. The NO synthesis activity was measured immediately once the samples were ready to use, and the substrate with cofactors was added immediately. The reactions were observed with Griess Reagents 1 and 2. The relative enzymatic activity of NOS was calculated with the absorbance at 540 nm.

### Nitric oxides (NO) measurement

To measure the total nitric oxides (NO), the HUAECs were incubated with VEGF, GSK591, or vehicle, and the supernatants were collected, followed by deproteinizing with a ten kDa column spin cut-off system (SARTORIUS, cat# VS0101). Then, the assay was performed immediately by the NO assay kit (Abcam, ab65328) with the Griess method according to the manufacturer’s protocol. To detect the NO production in living cells, the dye, 4-amino-5-methylamino-2′,7′-difluorofluorescein diacetate (DAF-FM DA; Invitrogen, cat# D23844) was used. The HUVECs were pretreated with DAF-FM DA for 15 min under indicated conditions, and the cells were washed for 10 min. The images were analyzed with a confocal microscopy system (LSM700, Zeiss), and the mean fluorescence intensity (MFI) was analyzed.

### Statistical analysis

All experiments were performed in triplicate under identical conditions, and the data were shown as means ± SEM. Unpaired two-tailed Student’s *t*-test analyzed differences between the two groups. The difference with *P* < 0.05 was considered statistically significant.

### Data availability statement

The data that support the findings of this study are available from the corresponding author upon reasonable request.

## References

[1] van Meerbeeck JP, Fennell DA, De Ruysscher DK. Small-cell lung cancer. Lancet. 2011; 378:1741–55. 10.1016/S0140-6736(11)60165-7 21565397

[2] Duma N, Santana-Davila R, Molina JR. Non-Small Cell Lung Cancer: Epidemiology, Screening, Diagnosis, and Treatment. Mayo Clin Proc. 2019; 94:1623–40. 10.1016/j.mayocp.2019.01.013 31378236

[3] Thai AA, Solomon BJ, Sequist LV, Gainor JF, Heist RS. Lung cancer. Lancet. 2021; 398:535–54. 10.1016/S0140-6736(21)00312-3 34273294

[4] Liu C, Qin Q, Cong H. Research Progress on the Relationship Between Mitochondrial Deoxyguanosine Kinase and Apoptosis and Autophagy in Lung Adenocarcinoma Cells. Cancer Insight. 2023; 1:53–62. 10.58567/ci01010004

[5] Kretschmer M, Rüdiger D, Zahler S. Mechanical Aspects of Angiogenesis. Cancers (Basel). 2021; 13:4987. 10.3390/cancers13194987 34638470 PMC8508205

[6] Lv H, Liu B, Qin Y. Isosorbide mononitrate promotes angiogenesis in embryonic development of zebrafish. Genet Mol Biol. 2020; 43:20190233. 10.1590/1678-4685-GMB-2019-0233 32706844 PMC7380327

[7] Lugano R, Ramachandran M, Dimberg A. Tumor angiogenesis: causes, consequences, challenges and opportunities. Cell Mol Life Sci. 2020; 77:1745–70. 10.1007/s00018-019-03351-7 31690961 PMC7190605

[8] Yi M, Jiao D, Qin S, Chu Q, Wu K, Li A. Synergistic effect of immune checkpoint blockade and anti-angiogenesis in cancer treatment. Mol Cancer. 2019; 18:60. 10.1186/s12943-019-0974-6 30925919 PMC6441150

[9] Riondino S, Del Monte G, Fratangeli F, Guadagni F, Roselli M, Ferroni P. Anti-Angiogenic Drugs, Vascular Toxicity and Thromboembolism in Solid Cancer. Cardiovasc Hematol Agents Med Chem. 2017; 15:3–16. 10.2174/1871525715666170127101605 28137223

[10] Chen H, Li H, Shi W, Qin H, Zheng L. The roles of m6A RNA methylation modification in cancer stem cells: new opportunities for cancer suppression. Cancer Insight. 2023; 1:1–18. 10.58567/ci01020001

[11] Hwang JW, Cho Y, Bae GU, Kim SN, Kim YK. Protein arginine methyltransferases: promising targets for cancer therapy. Exp Mol Med. 2021; 53:788–808. 10.1038/s12276-021-00613-y 34006904 PMC8178397

[12] Huang L, Zhang XO, Rozen EJ, Sun X, Sallis B, Verdejo-Torres O, Wigglesworth K, Moon D, Huang T, Cavaretta JP, Wang G, Zhang L, Shohet JM, et al. PRMT5 activates AKT via methylation to promote tumor metastasis. Nat Commun. 2022; 13:3955. 10.1038/s41467-022-31645-1 35803962 PMC9270419

[13] Yan Y, Zhao P, Wang Z, Liu Z, Wang Z, Zhang J, Ding Y, Hua X, Yu L. PRMT5 regulates colorectal cancer cell growth and EMT via EGFR/Akt/GSK3β signaling cascades. Aging (Albany NY). 2021; 13:4468–81. 10.18632/aging.202407 33495409 PMC7906165

[14] Lattouf H, Kassem L, Jacquemetton J, Choucair A, Poulard C, Trédan O, Corbo L, Diab-Assaf M, Hussein N, Treilleux I, Le Romancer M. LKB1 regulates PRMT5 activity in breast cancer. Int J Cancer. 2019; 144:595–606. 10.1002/ijc.31909 30289978 PMC6294691

[15] Huang J, Zheng Y, Zheng X, Qian B, Yin Q, Lu J, Lei H. PRMT5 Promotes EMT Through Regulating Akt Activity in Human Lung Cancer. Cell Transplant. 2021; 30:9636897211001772. 10.1177/09636897211001772 33829865 PMC8040599

[16] Beketova E, Owens JL, Asberry AM, Hu CD. PRMT5: a putative oncogene and therapeutic target in prostate cancer. Cancer Gene Ther. 2022; 29:264–76. 10.1038/s41417-021-00327-3 33854218 PMC8514585

[17] Liu M, Yao B, Gui T, Guo C, Wu X, Li J, Ma L, Deng Y, Xu P, Wang Y, Yang D, Li Q, Zeng X, et al. PRMT5-dependent transcriptional repression of c-Myc target genes promotes gastric cancer progression. Theranostics. 2020; 10:4437–52. 10.7150/thno.42047 32292506 PMC7150477

[18] Yuan Y, Nie H. Protein arginine methyltransferase 5: a potential cancer therapeutic target. Cell Oncol (Dordr). 2021; 44:33–44. 10.1007/s13402-020-00577-7 33469838 PMC12980669

[19] Kim H, Ronai ZA. PRMT5 function and targeting in cancer. Cell Stress. 2020; 4:199–215. 10.15698/cst2020.08.228 32743345 PMC7380451

[20] Tam SY, Wu VWC, Law HK. Hypoxia-Induced Epithelial-Mesenchymal Transition in Cancers: HIF-1α and Beyond. Front Oncol. 2020; 10:486. 10.3389/fonc.2020.00486 32322559 PMC7156534

[21] Karimi Roshan M, Soltani A, Soleimani A, Rezaie Kahkhaie K, Afshari AR, Soukhtanloo M. Role of AKT and mTOR signaling pathways in the induction of epithelial-mesenchymal transition (EMT) process. Biochimie. 2019; 165:229–34. 10.1016/j.biochi.2019.08.003 31401189

[22] Caniuguir A, Krause BJ, Hernandez C, Uauy R, Casanello P. Markers of early endothelial dysfunction in intrauterine growth restriction-derived human umbilical vein endothelial cells revealed by 2D-DIGE and mass spectrometry analyses. Placenta. 2016; 41:14–26. 10.1016/j.placenta.2016.02.016 27208404

[23] Boerma M, Burton GR, Wang J, Fink LM, McGehee RE Jr, Hauer-Jensen M. Comparative expression profiling in primary and immortalized endothelial cells: changes in gene expression in response to hydroxy methylglutaryl-coenzyme A reductase inhibition. Blood Coagul Fibrinolysis. 2006; 17:173–80. 10.1097/01.mbc.0000220237.99843.a1 16575254

[24] Mennerich D, Kubaichuk K, Kietzmann T. DUBs, Hypoxia, and Cancer. Trends Cancer. 2019; 5:632–53. 10.1016/j.trecan.2019.08.005 31706510

[25] Wicks EE, Semenza GL. Hypoxia-inducible factors: cancer progression and clinical translation. J Clin Invest. 2022; 132:e159839. 10.1172/JCI159839 35642641 PMC9151701

[26] Popper HH. Progression and metastasis of lung cancer. Cancer Metastasis Rev. 2016; 35:75–91. 10.1007/s10555-016-9618-0 27018053 PMC4821869

[27] Ridnour LA, Isenberg JS, Espey MG, Thomas DD, Roberts DD, Wink DA. Nitric oxide regulates angiogenesis through a functional switch involving thrombospondin-1. Proc Natl Acad Sci USA. 2005; 102:13147–52. 10.1073/pnas.0502979102 16141331 PMC1201580

[28] Hirota K, Semenza GL. Regulation of angiogenesis by hypoxia-inducible factor 1. Crit Rev Oncol Hematol. 2006; 59:15–26. 10.1016/j.critrevonc.2005.12.003 16716598

[29] Fantozzi A, Gruber DC, Pisarsky L, Heck C, Kunita A, Yilmaz M, Meyer-Schaller N, Cornille K, Hopfer U, Bentires-Alj M, Christofori G. VEGF-mediated angiogenesis links EMT-induced cancer stemness to tumor initiation. Cancer Res. 2014; 74:1566–75. 10.1158/0008-5472.CAN-13-1641 24413534

[30] Yang Y, Bedford MT. Protein arginine methyltransferases and cancer. Nat Rev Cancer. 2013; 13:37–50. 10.1038/nrc3409 23235912

[31] Feustel K, Falchook GS. Protein Arginine Methyltransferase 5 (PRMT5) Inhibitors in Oncology Clinical Trials: A review. J Immunother Precis Oncol. 2022; 5:58–67. 10.36401/JIPO-22-1 36034581 PMC9390703

[32] Kim H, Kim H, Feng Y, Li Y, Tamiya H, Tocci S, Ronai ZA. PRMT5 control of cGAS/STING and NLRC5 pathways defines melanoma response to antitumor immunity. Sci Transl Med. 2020; 12:eaaz5683. 10.1126/scitranslmed.aaz5683 32641491 PMC7508354

[33] Jing P, Zhao N, Ye M, Zhang Y, Zhang Z, Sun J, Wang Z, Zhang J, Gu Z. Protein arginine methyltransferase 5 promotes lung cancer metastasis via the epigenetic regulation of miR-99 family/FGFR3 signaling. Cancer Lett. 2018; 427:38–48. 10.1016/j.canlet.2018.04.019 29679612

[34] Zhang S, Ma Y, Hu X, Zheng Y, Chen X. Targeting PRMT5/Akt signalling axis prevents human lung cancer cell growth. J Cell Mol Med. 2019; 23:1333–42. 10.1111/jcmm.14036 30461193 PMC6349228

[35] Rakotomalala A, Escande A, Furlan A, Meignan S, Lartigau E. Hypoxia in Solid Tumors: How Low Oxygenation Impacts the “Six Rs” of Radiotherapy. Front Endocrinol (Lausanne). 2021; 12:742215. 10.3389/fendo.2021.742215 34539584 PMC8445158

[36] Cairns RA, Papandreou I, Sutphin PD, Denko NC. Metabolic targeting of hypoxia and HIF1 in solid tumors can enhance cytotoxic chemotherapy. Proc Natl Acad Sci USA. 2007; 104:9445–50. 10.1073/pnas.0611662104 17517659 PMC1890514

[37] Muz B, de la Puente P, Azab F, Azab AK. The role of hypoxia in cancer progression, angiogenesis, metastasis, and resistance to therapy. Hypoxia (Auckl). 2015; 3:83–92. 10.2147/HP.S93413 27774485 PMC5045092

[38] Arriagada C, Silva P, Torres VA. Role of glycosylation in hypoxia-driven cell migration and invasion. Cell Adh Migr. 2019; 13:13–22. 10.1080/19336918.2018.1491234 30015560 PMC6527385

[39] Hoffmann C, Mao X, Brown-Clay J, Moreau F, Al Absi A, Wurzer H, Sousa B, Schmitt F, Berchem G, Janji B, Thomas C. Hypoxia promotes breast cancer cell invasion through HIF-1α-mediated up-regulation of the invadopodial actin bundling protein CSRP2. Sci Rep. 2018; 8:10191. 10.1038/s41598-018-28637-x 29976963 PMC6033879

[40] Emami Nejad A, Najafgholian S, Rostami A, Sistani A, Shojaeifar S, Esparvarinha M, Nedaeinia R, Haghjooy Javanmard S, Taherian M, Ahmadlou M, Salehi R, Sadeghi B, Manian M. The role of hypoxia in the tumor microenvironment and development of cancer stem cell: a novel approach to developing treatment. Cancer Cell Int. 2021; 21:62. 10.1186/s12935-020-01719-5 33472628 PMC7816485

[41] Tong WW, Tong GH, Liu Y. Cancer stem cells and hypoxia-inducible factors (Review). Int J Oncol. 2018; 53:469–76. 10.3892/ijo.2018.4417 29845228

[42] Cao D, Hou M, Guan YS, Jiang M, Yang Y, Gou HF. Expression of HIF-1alpha and VEGF in colorectal cancer: association with clinical outcomes and prognostic implications. BMC Cancer. 2009; 9:432. 10.1186/1471-2407-9-432 20003271 PMC2797529

[43] Zeng Z, Huang WD, Gao Q, Su ML, Yang YF, Liu ZC, Zhu BH. Arnebin-1 promotes angiogenesis by inducing eNOS, VEGF and HIF-1α expression through the PI3K-dependent pathway. Int J Mol Med. 2015; 36:685–97. 10.3892/ijmm.2015.2292 26202335 PMC4533782

[44] Vimalraj S, Bhuvaneswari S, Lakshmikirupa S, Jyothsna G, Chatterjee S. Nitric oxide signaling regulates tumor-induced intussusceptive-like angiogenesis. Microvasc Res. 2018; 119:47–59. 10.1016/j.mvr.2018.04.001 29649432

[45] Lala PK, Orucevic A. Role of nitric oxide in tumor progression: lessons from experimental tumors. Cancer Metastasis Rev. 1998; 17:91–106. 10.1023/a:1005960822365 9544425

[46] Park SJ, Yoon BH, Kim SK, Kim SY. GENT2: an updated gene expression database for normal and tumor tissues. BMC Med Genomics. 2019 (Suppl 5); 12:101. 10.1186/s12920-019-0514-7 31296229 PMC6624177

[47] Huang L, Liu J, Zhang XO, Sibley K, Najjar SM, Lee MM, Wu Q. Inhibition of protein arginine methyltransferase 5 enhances hepatic mitochondrial biogenesis. J Biol Chem. 2018; 293:10884–94. 10.1074/jbc.RA118.002377 29773653 PMC6052201

